# Understanding anesthesia anxiety: A mixed-methods analysis of propofol discourse on reddit

**DOI:** 10.1177/20552076261434056

**Published:** 2026-03-13

**Authors:** James R. Burmeister, John K. Jung, Ismail Zazay, Roy G. Soto

**Affiliations:** 1 Department of Foundational Medical Studies, 159878 Oakland University William Beaumont School of Medicine, Rochester, MI, USA; 2 John Sealy School of Medicine, 12338 University of Texas Medical Branch, Galveston, TX, USA; 3 Attending Anesthesiologist, Residency Program Director, Department of Anesthesiology, 21818 Corewell Health William Beaumont University Hospital, Royal Oak, MI, USA

**Keywords:** Propofol, anesthesia, anxiety, social media, natural language processing, health communication, patient participation

## Abstract

**Objective:**

Propofol is widely used in procedural sedation and general anesthesia, but often provokes anxiety among patients and some providers. This study investigates the emotional and thematic landscape of propofol-related discourse on Reddit, a major online health information platform.

**Methods:**

We analyzed 921 publicly available Reddit posts referencing “propofol” and related sedation terms using a mixed-methods approach. Sentiment analysis was performed with TextBlob and complemented by manual thematic coding. Posts were categorized by subreddit, sentiment, and topic. Descriptive statistics and correlation analyses examined relationships between sentiment, word count, and subreddit type.

**Results:**

Two coders achieved strong agreement (Cohen's κ = 0.82). Half of posts were neutral, whereas 30% were negative and 20% were positive. Negative sentiment was most common in patient-focused subreddits such as r/colonoscopy (38%), while provider forums like r/anesthesiology were more neutral or analytical. Among posts, 52% were patient-authored, 28% provider-authored, and 20% unclear. Patients more often expressed anxiety and confusion, while providers discussed clinical dilemmas and ethical issues. Higher word count was weakly correlated with more negative sentiment (*r* = −0.19). Four thematic clusters emerged: clinical sedation and medication questions; provider professionalism and ethics; veterinary use and animal care; and exam stress or career anxiety.

**Conclusion:**

Reddit reveals emotionally rich propofol discourse, spanning patient fears and provider uncertainties. Analysis using digital health frameworks such as affective publics and the Technology Acceptance Model highlights opportunities for improved patient communication, education, and digital tool design. Limitations include platform demographic bias and limited generalizability. These findings offer a methodological foundation and conceptual framework for future digital health research and sentiment-aware clinical tools.

## What this study adds

- Provides one of the first hybrid qualitative–computational analyses of propofol discourse on Reddit.
- Identifies key themes around patient anxiety, provider reflection, and sedation ethics.
- Demonstrates how digital health frameworks like affective publics and Technology Acceptance Model (TAM) can contextualize online anesthesia discussions.
- Offers ethical reflection on the use of pseudonymous health narratives in research.

## Introduction

Propofol, a short-acting intravenous anesthetic, is commonly used for the induction and maintenance of anesthesia and procedural sedation.^1^ Despite its routine use in clinical practice, understanding of propofol's effects and risks remains uneven across patient and provider groups.^1^ Few studies have explored patient or provider perceptions of anesthesia online; prior research has primarily focused on clinical outcomes or in-person experiences.

This study addresses this gap by analyzing authentic, user-generated discourse about propofol and anesthesia anxiety on Reddit, thereby expanding the literature on digital health information-seeking and affective publics, networked groups who mobilize around shared emotions and narratives in digital spaces.

Reddit, a pseudonymous discussion platform, has emerged as a source for unsolicited user-generated posts on medical experiences and dilemmas. Recent studies have begun to analyze anesthesia-related discourse on social media, highlighting both patient concerns and educational opportunities for providers.^2^ For example, Almeida et al. (2025) leveraged Reddit to identify medication experiences among patients with opioid use disorder, highlighting the platform's potential for uncovering nuanced, patient-driven health concerns.^3^ Similarly, Jain et al. (2024) emphasized the increasing value of social media as a tool for anesthesia education and patient engagement.^2^ These studies reinforce the importance of digital health research for capturing the affective and cognitive dimensions of medical experience online. Connell (2025) also notes key demographic characteristics of Reddit's user base, which is important context for interpreting digital health findings.^4^ Our study builds on this evolving literature by focusing specifically on propofol-related anxiety and by integrating sentiment analysis with thematic coding in a digital health framework.

In this study, we explore how both patients and healthcare professionals discuss propofol, focusing on sentiment, emerging themes, and the broader emotional landscape of online anesthesia discourse. Propofol is associated not only with sedation but also with patient fears of loss of control, amnesia, intraoperative awareness, and, in rare cases, abuse or misuse. These anxieties often motivate patients and providers to seek or share information in online forums.^2^

We situate our analysis within digital health theory, considering how affective publics and online health information-seeking behaviors shape discourse around medical interventions. By employing a hybrid qualitative–computational approach within digital health theoretical frameworks, this study not only explores Reddit discourse but also establishes a scalable model for future cross-platform analyses of patient and provider sentiment around anesthesia and procedural sedation. Our findings offer a foundation for advancing research on digital health communication, patient–clinician dialogue, and the ethical use of online data in health services.

## Methods

This is a cross-sectional, mixed-methods study. The dataset comprises 921 publicly available Reddit posts referencing “propofol,” collected between January and June 2025 via manual search. All data were collected online. All posts are in publicly available subreddits including r/colonoscopy, r/nursing, r/medicine, r/AskDocs, r/medicalschool, r/Residency, r/veterinary and r/anesthesiology. Posts included original questions, clinical reflections, and peer commentary. Posts were identified via manual keyword search and included both original posts and comments, after excluding off-topic content, jokes, or spam. Only English-language posts were included; non-English posts were excluded from analysis. To determine post authorship (patient, provider, or unclear), we applied a manual coding protocol. Posts were attributed as “patient-authored” if they appeared in patient-focused subreddits, such as r/colonoscopy and r/AskDocs, or included first-person narratives about personal experience with sedation. Posts in provider-oriented subreddits such as r/anesthesiology, /medicalschool, and r/nursing, or those containing language about clinical duties, medical training, or ethical dilemmas in care, were coded as “provider-authored.” Posts with ambiguous language or insufficient context for attribution were labeled “unclear.” Both coders independently classified each post's likely authorship, and disagreements were resolved by consensus.

To ensure consistency in manual coding, two coders were trained on a sample dataset and later independently labeled all posts for sentiment and thematic group. Disagreements were resolved through consensus discussion, and Cohen's kappa was calculated to assess reliability. Sentiment was analyzed using TextBlob, a lexicon-based NLP tool. Themes were derived inductively through iterative coding and comparison.

We compared sentiment distribution across subreddits and calculated descriptive statistics (upvotes, word count, correlation coefficients) to explore relationships between post features and sentiment. Thematic clusters were derived through iterative reading and comparative coding. Representative quotes were extracted to illustrate common concerns and tone.

## Results

**Figure 1.**
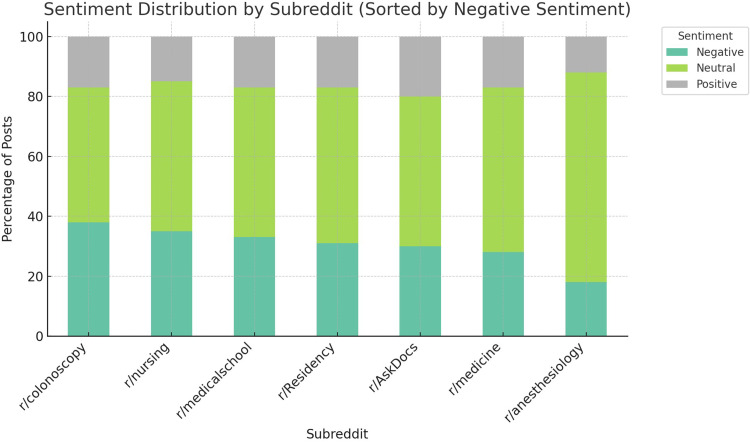
Sentiment distribution by subreddit. Bar chart showing the percentage of posts with positive, neutral, and negative sentiment across key subreddits (e.g. r/colonoscopy, r/nursing, r/anesthesiology, r/veterinary, and r/medicalschool). Patient-centered forums had a higher proportion of negative sentiment, while professional forums leaned neutral or analytical.

Cohen's kappa was calculated at 0.82, indicating a high level of coder agreement. Of the 921 posts analyzed, 50% (*n* = 461) were neutral, 30% (*n* = 276) negative, and 20% (*n* = 184) positive. Posts from patient-focused subreddits like r/colonoscopy showed a higher proportion of negative sentiment (38%), whereas professional subreddits such as r/anesthesiology had more neutral or analytical tone. Posts with higher word counts were more likely to be negative, and sentiment did not strongly correlate with upvote count. Negative posts commonly conveyed procedural anxiety, mistrust of sedation, or ethical concerns over medication use. Thematic categorization revealed four core clusters (Figure 1):

1. **Clinical Sedation and Medication Questions**: Topics included dosage clarification, propofol combinations, and patient confusion. Example: “According to UBP you can give 20 mg prop for PO case without MAC if surgeon requests. Anyone confirm?”
2. **Nurse and Provider Professionalism**: New providers and nurses shared dilemmas involving unclear orders, scope of practice, and workplace stress. Example: “Will I get in trouble for administering propofol when the doctor wasn’t clear in the orders?”
3. **Veterinary and Rescue Contexts**: A small subset focused on the emotional and clinical use of propofol in end-of-life care for pets. Posts often urged immediate action to prevent euthanasia.
4. **Exam Preparation and Career Anxiety**: Posts within r/anesthesiology and similar subreddits reflected on test strategies, drug memorization, and the pressure to perform. Example: “Other than doing TrueLearn, what are high performers doing for BASIC prep?”

These themes reflect both patient-facing concerns and behind-the-scenes provider deliberations. High-engagement posts (receiving 40–130+ upvotes) often blended practical knowledge with emotional vulnerability.

**Figure 2.**
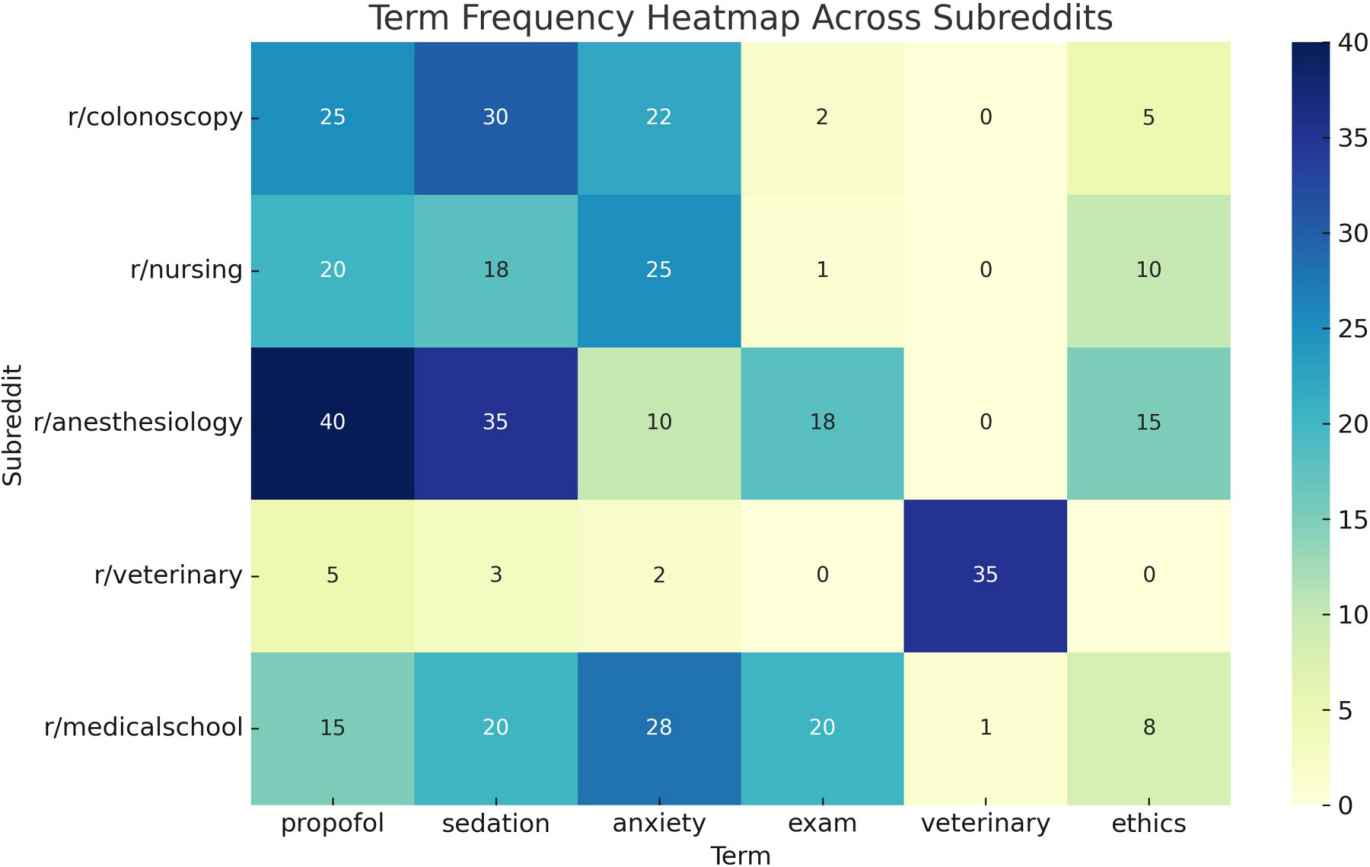
Term frequency heatmap across subreddits. Heatmap displaying the frequency of key terms (e.g. “propofol,” “anxiety,” “ethics,” “exam”) across subreddit categories. Each cell reflects how often a term appeared in a specific subreddit, illustrating topic emphasis and thematic divergence between user groups.

As shown in Figure 2, term usage varied significantly across subreddits: “propofol” and “sedation” were most frequent in r/anesthesiology and r/colonoscopy, while “anxiety” appeared prominently in r/nursing and r/medicalschool. “Exam” dominated r/medicalschool, and “veterinary” was almost exclusive to r/veterinary.

## Discussion

**Figure 3.**
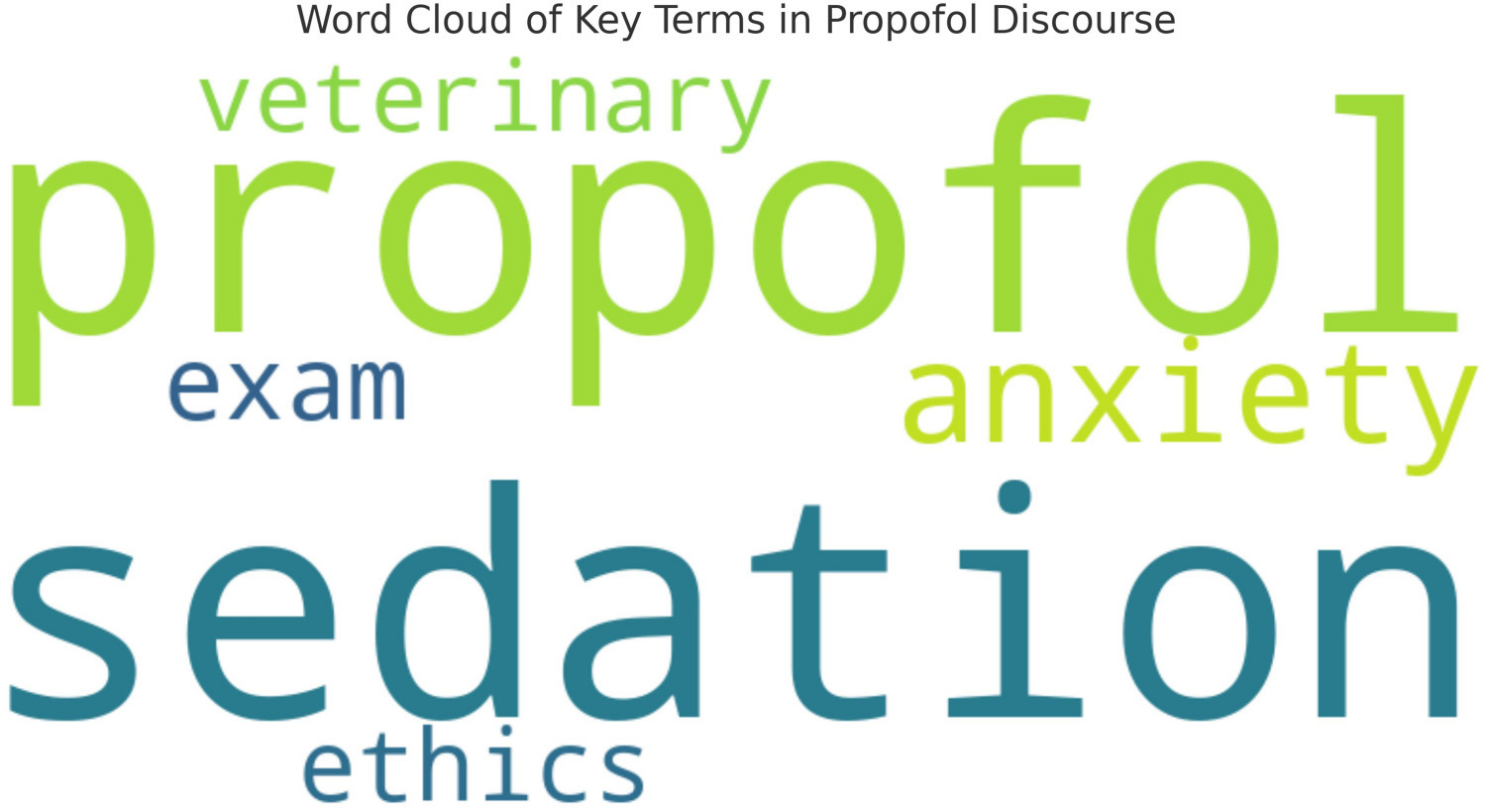
Word cloud of key terms in propofol discourse. Word cloud generated from the entire Reddit dataset, showing the most frequently used terms in discussions mentioning propofol. Larger words represent higher frequency. Terms like “sedation,” “anxiety,” “dosage,” and “exam” indicate central concerns in the dataset.

To summarize common lexical trends across posts, we generated a word cloud from the full Reddit corpus (Figure 3). Figure 3 displays the most common terms in Reddit posts about propofol, with “sedation,” “propofol,” “anxiety,” “exam,” and “ethics” appearing most frequently, highlighting dominant themes of clinical use, emotional concern, and ethical reflection.

This analysis reveals a multi-layered conversation about propofol, spanning patient anxiety, professional uncertainty, and knowledge-sharing within medical communities. Online platforms like Reddit provide insight into how both patients and providers make sense of complex pharmacologic tools.^5^ The use of a hybrid manual and computational approach adds interpretive richness and methodological transparency. This work contributes to the growing body of digital health scholarship that examines affective publics, emotionally charged collectives formed around health concerns, and highlights online health information-seeking behavior as a key driver of sentiment. The TAM and Health Belief Model offer additional frameworks through which Reddit users’ emotional and cognitive appraisals of sedation may be understood, especially regarding perceived risk, trust in information sources, and perceived benefits of undergoing or refusing sedation.^6^ This work moves beyond descriptive findings by demonstrating a reproducible approach to synthesizing sentiment and thematic analysis in digital health research, thereby supporting the development of patient-centered education, clinical decision support, and future studies using advanced computational models.

While our sample size supports robust thematic and sentiment analysis, generalizability remains limited by platform-specific bias. Reddit's user base is younger, predominantly male, and technologically literate, which may introduce selection bias.^4^ Reddit is a valuable platform for exploring user-generated health discourse; however, it is important to note that users who post about propofol are a self-selected subset of the general population undergoing procedural sedation. Also, individuals who post online about their experiences may differ systematically from those who do not, for example, by having greater anxiety, unique experiences, or more interest in medical topics. Therefore, findings from Reddit cannot be assumed to represent the full spectrum of patients or providers exposed to propofol in clinical settings. Moreover, Reddit posts lack demographic metadata and are subject to deletion, moderation, and community-specific norms that can shape visibility and tone.^7^ These factors constrain extrapolation but offer unique insight into how specific anxieties and clinical decision points are articulated in digitally native spaces.^8^

For human-coded sentiment, we selected TextBlob for its transparency and ease of validation but acknowledge that lexicon-based tools have recognized limitations in handling clinical nuance, sarcasm, and informal language. To partially address this, we cross-validated TextBlob sentiment assignments on a randomly selected subsample (*n* = 100) using VADER, a sentiment tool optimized for social media, finding comparable distributions. We did not apply VADER to the full dataset due to computational and feasibility constraints, prioritizing a mixed-methods, reproducible workflow in line with our study aims. Future work should incorporate transformer-based models such as BERT and RoBERTa for deeper semantic analysis.^5,8^ Our study was exploratory in nature; future modeling efforts will compare transformer-based sentiment tools to better assess topic modeling and emotion extraction across clinical themes.

Clinicians can leverage these findings to tailor preoperative counseling, addressing common anxieties identified online and using patient-centered language to improve understanding and reduce fear. Incorporating online-derived concerns into patient education materials and shared decision-making discussions may further enhance patient trust and satisfaction. Future research should apply advanced sentiment analysis models such as transformer-based tools like BERT or RoBERTa and compare sentiment across multiple platforms beyond Reddit to increase generalizability and analytic depth. In summary, our mixed-methods approach offers a transferable model for digital health researchers seeking to combine qualitative insight with computational rigor in the analysis of online medical discourse. The frameworks and findings described here can inform future research and practical interventions to address anesthesia-related anxiety in clinical and digital environments.

## Limitations

This study has several limitations. First, our analysis is limited to a single platform (Reddit), which is not representative of the broader patient or provider population. Reddit users are predominantly younger, male, and technologically literate, which may introduce selection bias.^4^ Our sample of 921 posts, while sufficient for qualitative and exploratory computational analysis, may not capture the full range of perspectives or experiences with propofol-related anxiety. Thus, findings should be interpreted as reflective of a specific online demographic rather than generalizable to all individuals undergoing anesthesia. The use of a single data source and moderate sample size constrains external validity and should be addressed in future multi-platform or larger-scale studies.

Second, sentiment and thematic analysis are subject to coder interpretation and may be influenced by platform-specific language or sarcasm, which can challenge accurate polarity detection. Third, although we achieved strong intercoder reliability, there is potential for misclassification in both sentiment and theme assignment. Fourth, the use of a single platform (Reddit) restricts generalizability; findings may not apply to discussions on other social media or patient forums. Finally, posts may be deleted or moderated after initial collection, limiting data reproducibility. Our dataset reflects only the perspectives of users who actively choose to share anesthesia-related experiences on Reddit. This self-selection introduces further bias, as these users may have stronger emotions, unusual experiences, or higher digital literacy than typical patients.

Further, our reliance on TextBlob for sentiment analysis introduces known limitations. Lexicon-based tools often misclassify nuanced or sarcastic language, which is prevalent in Reddit discourse, and may fail to capture complex medical sentiment. Although we partially mitigated this by comparing a subsample with VADER, future studies should apply advanced models such as BERT or RoBERTa to improve sensitivity to domain-specific language and humor.

## Conclusion

These insights have potential implications for health communication, informatics, and education. For clinicians, understanding the emotional narratives surrounding sedation can improve patient counseling and shared decision-making.^9^ Anxiety flagged in online forums may indicate where additional preoperative education, anticipatory guidance, or empathic framing are most needed. For informatics developers, sentiment-tagged corpora like this one may inform chatbot logic or decision support features in anesthesia risk apps. In clinical education, Reddit discourse reveals real-world challenges faced by nursing and anesthesia trainees, offering cases that may enrich simulation or ethics curricula. These findings could also inform collaborations between clinicians and platform moderators to identify anxiety hotspots or improve the accuracy of sedation-related community health threads.

Discussions about propofol on Reddit reveal a wide range of sentiment and concerns across patient, provider, and veterinary contexts. From fear and confusion to career stress and ethical dilemmas, these online narratives underscore the need for accessible and accurate education around anesthesia practices. Reddit serves as both a mirror of public sentiment and a potential tool for identifying misinformation and emotional strain among healthcare workers. Grounding our findings in digital health frameworks enhances the applicability of this research to ongoing work in health communication, risk perception, and emotional public engagement. A visual representation of sentiment by subreddit (included in Figure 1) further illustrates the stratified emotional landscape of propofol discourse. As digital platforms increasingly shape how patients and providers share, seek, and interpret health information, understanding the emotional topography of anesthesia discourse online can inform more empathetic care, better health communication, and smarter digital tool design.
